# Differential response of tomato genotypes to *Xanthomonas*-specific pathogen-associated molecular patterns and correlation with bacterial spot (*Xanthomonas perforans*) resistance

**DOI:** 10.1038/hortres.2016.35

**Published:** 2016-08-10

**Authors:** Krishna Bhattarai, Frank J Louws, John D Williamson, Dilip R Panthee

**Affiliations:** 1 Department of Horticultural Science, North Carolina State University, Mountain Horticultural Crops Research and Extension Center, Mills River, Mills River, NC 28759, USA; 2 Department of Plant Pathology, North Carolina State University, Raleigh, NC 27695, USA; 3 Department of Horticultural Science, North Carolina State University, Raleigh, NC 27695, USA

## Abstract

Plants depend on innate immune responses to retard the initial spread of pathogens entering through stomata, hydathodes or injuries. These responses are triggered by conserved patterns in pathogen-encoded molecules known as pathogen-associated molecular patterns (PAMPs). Production of reactive oxygen species (ROS) is one of the first responses, and the resulting ‘oxidative burst’ is considered to be a first line of defense. In this study, we conducted association analyses between ROS production and bacterial spot (BS; *Xanthomonas* spp.) resistance in 63 genotypes of tomato (*Solanum lycopersicum* L.). A luminol-based assay was performed on leaf tissues that had been treated with a flagellin 22 (flg22), flagellin 28 and a *Xanthomonas-*specific flg22 (flg22*-Xac*) peptide, to measure PAMP-induced ROS production in each genotype. These genotypes were also assessed for BS disease response by inoculation with *Xanthomonas perforans*, race T4. Although there was no consistent relationship between peptides used and host response to the BS, there was a significant negative correlation (*r*=−0.25, *P*<0.05) between foliar disease severity and ROS production, when flg22*-Xac* was used. This response could potentially be used to identify the *Xanthomonas*-specific PRR allele in tomato, and eventually PAMP-triggered immunity loci could be mapped in a segregating population. This has potential significance in tomato improvement.

## Introduction

Tomato (*Solanum lycopersicum* L.) is the second most important vegetable crop in the world, as well as being an important model plant for genetics and genomics studies because of its relatively short reproductive cycle and small genome. Bacterial spot (BS) caused by four species of *Xanthomonas*: *X. euvesicatoria*, *X. vesicatoria*, *X. perforans* and *X. gardneri* ^1^ has become a global problem in tomato causing severe yield losses.

Five races (T1–T5) of *Xanthomonas* have been recognized, based on differential hypersensitive responses on different tomato genotypes.^1^ To complicate breeding efforts, alteration and/or migration of virulent strains can occur, overcoming available resistance to existing races, even before novel resistance can be deployed. Further, developing durable genetic resistance against BS has been a challenge due to its multigenic nature and the limited availability of resistance genes in tomato accessions.

Foliar bacterial plant pathogens enter plant tissues through pre-existing openings such as stomata, hydathodes or injuries. Unlike animals, plants do not have an adaptive immune system and thus depend on preformed defenses or innate immunity, and induced responses to infection.^2–4^ Plants are able to identify highly conserved pathogen molecules, pathogen-associated molecular patterns (PAMPs), that are present in pathogen structures and that are necessary for the survival and infectivity of the pathogen, but are absent in the host plant.^2,5^ PAMPs in bacteria include flagellins (flg), lipopolysaccharides, cold-shock proteins (csp), peptidoglycans and ‘elongation factor thermal unstable’ (EF-Tu) proteins.^6,7^ Plant immunity relies on the ability to identify invading microbes by means of endogenous molecular patterns; when these are the result of tissue damage these are called damage-associated molecular patterns (DAMPs).^8^ Identification of PAMPs or DAMPs by the plant triggers initiation of defense responses known as either PAMP-triggered immunity (PTI) or DAMP-triggered immunity, respectively.^8–11^ In response, pathogens have evolved mechanisms to evade PTI by releasing effectors into the plant cell that suppress host resistance responses and permit pathogen infection. Plants have, in turn, evolved mechanisms to identify and block the effect of these specific effectors and thus limit infection. Together, this interaction is known as effector-triggered immunity,^3,12^ which is more effective than PTI. Recognition of PAMPs by surface-localized plasma membrane pattern recognition repeat (PRR) leads to a signaling cascade initiating PTI.^7^ PTI responses include production of reactive oxygen species (ROS), protein phosphorylation, ethylene biosynthesis and cell wall reinforcement by callose deposition.^11,13–16^

ROS are generated by excitation or incomplete reduction of molecular oxygen during cellular metabolism in aerobic organisms.^17,18^ These reactive intermediates can be produced when molecular dioxygen (O_2_) accepts electrons released by various reactions in the cell. Superoxide (O_2_^·−^) and hydrogen peroxide (H_2_O_2_) are two major ROS found in tomato. The plasmalemma-localized NAD(P)H oxidases and apoplastic superoxide dismutases (SODs) are the main producers of O_2_^·−^ and H_2_O_2_, respectively,^18^ in the extracellular matrix. This rapid elicitation of ROS production by host cells after recognition of PAMPs is known as the oxidative burst, and the ROS generated can be both anti-microbial and signals for induction of other defense responses.^5^

Perception of PAMPs such as the flagellin peptides initiates ROS production in plant cells. The most studied bacterial PAMPs are flagellin 22 (flg22)^19^ and flagellin 28 (flgII-28)^20^ synthesized based on a flagellin sequences from *Pseudomonas syringae* pv. *tomato*, and csp22 synthesized based on cold-shock protein (csp) as described by Felix *et al*.^21^

Identification of PAMP recognition specificity across plant families could provide a powerful strategy for developing resistance to a wide range of pathogens. For instance, transfer of elongation factor receptor kinase (EFR), a pattern recognition receptor (PRR) from the cruciferous plant *A. thaliana* that confers responsiveness to bacterial EF-Tu,^7,11,16^ provides increased resistance in a number of solanaceous crops such as *Nicotiana benthamiana* and *S. lycopersicum* to a number of genera of phytopathogenic bacteria. Exploiting PRR recognition specificity and associated host defense signaling pathways has several advantages over current disease management practices as well as current methods for developing disease resistance. Exploiting the innate immunity of plants to control pathogens could lead to a more durable source of resistance, decrease use of pesticides and could reduce the financial, human health and environmental costs associated with managing plant diseases.

It is hypothesized that pathogen mutations that overcome PAMP-mediated resistance would occur at low frequency, as the molecule recognized by PRRs is frequently associated with the fitness of the pathogen. In addition to potentially being more durable, the ability to rapidly transfer new PRRs to elite crop varieties through genetic transformation could expedite resistance breeding.^8,22–24^ Proceeding towards this goal, ROS production in *Arabidopsis* and tomato leaf tissue in response to live *P. syringae* pv *tomato* strains as well as to synthesized peptide sequences were previously assessed.^25^ The potential of using PAMPs to correlate the extent of PTI responsiveness to quantitative resistance against distinct pathogens has been previously reported.^15,16^ The range of ROS responses of an F2 tomato population to different peptides has also been reported.^25^

Although bacterial flagellins are perceived by all solanaceous crops, the response to different peptide sequences in the protein has been reported to vary.^26,27^ In the present study, we used an allelic variant of the *P. aeruginosa* flg22, derived from *Xanthomonas axonopodis* pv. *citri* flagella, flg22^*Xac*^, henceforth referred to as *Xanthomonas-*specific flg22 (flg22*-Xac*). This PAMP is known to elicit detectable ROS production in *Arabidopsis thaliana*. Here we evaluate the induction of ROS production in response to flg22*-Xac* along with flg22 and flgII-28, and assess the association of ROS production with BS disease resistance in a large number of tomato genotypes, derived from a wide range of genetic backgrounds.

## Materials and methods

### Plant materials and growth conditions

Sixty-three tomato genotypes including lines from tomato breeding programs at North Carolina State University and the University of Florida, as well as heirloom and wild lines were assessed. Seeds were sown in 4P soil mixture (Fafard, Florida, USA) in 24-cell trays for the greenhouse experiment in March 2013 at the Method Road Greenhouse, North Carolina State University, Raleigh, NC, USA. Six plants per genotype were planted in three replications, and the experiment was conducted in a completely randomized design. Plants in the greenhouse study were fertilized using a 20:20:20 ratio of nitrogen, phosphorus and potassium, respectively. Standard greenhouse pesticide application was used for insect and fungal disease (powdery mildew) control. For the field experiment, the same 63 genotypes were sown in flat bed metal trays in a standard seeding mix (2:2:1, v/v/v) peat moss:pine bark:vermiculite with macro- and micronutrients (Van Wingerden International Inc., Mills River, NC, USA) in May 2013. After 10 days, seedlings were transplanted to 72-cell flats (56×28 cm). After 4 weeks, these plants were transplanted to the field at the Mountain Horticultural Crops Research and Extension Center, Mills River, NC, USA. Six plants per plot were planted with a plant to plant spacing of 45 cm and with 150 cm between rows in two replications in a randomized complete block design. Standard management practices for fertilization, insect management and management of foliar fungal diseases were used.^28^ Neither copper nor Actigard products, commonly used in tomato production to suppress bacterial diseases, were applied in any experiments.

### ROS assay: preparation of reagents

Polypeptides designed from *P. syringae* pv. tomato flg22,^19^ flgII-28^20^ and flg22*-Xac*^29^ were used in this experiment. The amino-acid sequence of the flg22*-Xac* was QRLSSGLRINSAKDDAAGLAIS, flg22 was QRLSTGSRINSAKDDAAGLQIA and flgII-28 was ESTNILQRMRELAVQSRNDSNSATDREA (EZBiolabs, Carmel, IN, USA) as described above. Peptides were dissolved in distilled water (dH_2_O) at a concentration of 100 nm for use. Luminol (Sigma Lifescience, Saint Louis, MO, USA) was dissolved in dimethyl sulfoxide at a concentration of 17 mg mL^−1^, in the dark. Horseradish peroxidase (Sigma Type VI-A, Saint Louis, MO, USA) was dissolved in dH_2_O at a concentration of 10 mg m^−1^.

### Preparation of samples

Four millimeter diameter leaf disks were excised from the fully expanded leaf of each plant, second from the top, using a cork borer. Leaf disks were incubated with the adaxial side up in 200 μL Lumitrac 200 medium 96-well microplate (Greiner Bio-One, Inc., Monroe, NC, USA, product #: 655075) for 16–24 h at room temperature covered with aluminum foil. Four leaf disks from each tomato line were sampled for each experiment from greenhouse as well as field as described above for ROS assay.

The incubation media was removed from each well and replaced with 100 μl of a solution containing 12 μl of peptide solution, 24 μl of horseradish peroxidase and 24 μl of luminol diluted in 12 ml of dH_2_O, and measurement of photon productions in the form of relative light units assessed. Preparation of the mixture containing peptide, horseradish peroxidase and luminol, as well as the addition of ingredients to the samples was performed in the dark to avoid potential photodegradation.

### Measurement of ROS production

Production of ROS in each sample was measured as photon production quantitated as relative light units (RLUs) using a Glomax 96 microplate luminometer (Promega Corporation, Madison, WI, USA) for the greenhouse experiment, and a Biotek Multi Detection Microplate Reader Synergy 2 for the field experiment. Photon production was measured over 15 cycles at 4 min intervals for a total of 60 min.

Negative controls for luminol assays consisted of incubating leaf tissues from several arbitrarily selected lines in 200 μl of dH_2_O in 96-well plates. After 16 h, the dH_2_O was removed and 100 μl of assay solution including 2 μl of luminol and horseradish peroxidase but no peptide was added to each well.

### Inoculum preparation and inoculation

Plants in both greenhouse and field studies were inoculated with a preparation of field isolate 9 collected from infected tissue of tomato plants from a field in North Carolina that was subsequently characterized as *X. perforans* race T4 (by the Dr Jefferey B. Jones lab, University of Florida, Gainesville, FL, USA). This isolate was collected from private tomato fields as well as research plots at Mountain Horticultural Crops Research & Extension Center, Mills River, NC, USA. Although no specific permissions were required to collect samples from Mountain Horticultural Crops Research & Extension Center, permissions were obtained from the growers before going to the private fields. This strain was grown in pure culture from a single colony-forming unit and stored at −80 °C. To prepare inoculum, frozen stock was revived on yeast dextrose chalk agar medium^30^ and incubated for 24–48 h at 30 °C. Plates were then flooded with distilled water and bacterial cells were scrapped with a wire loop and recovered in suspension. Bacterial density was measured as OD_600_ on a LKB Biochrom Ultrospec II spectrophotometer (American Laboratory Trading, Inc., Cambridge, MA, USA) and inoculum prepared by adjusting the bacterial concentration to 0.3 OD_600_ (~2–5×10^8^ colony-forming unit per mL). This freshly prepared suspension was immediately used for inoculations.

For greenhouse inoculations, humidity in the immediate vicinity of the plants was maintained using V5100NS humidifiers (Vicks Ultrasonic Humidifiers, NY, USA) from 24 h prior to the inoculation to 48 h after inoculation and covering plants with white plastic. Spray inoculation to foliar runoff was performed 30 days after sowing using a hand sprayer. In the field, uniform spray inoculation was performed using a backpack sprayer 30 days after transplanting.

### Disease evaluation

Greenhouse plants were scored for symptoms on the most severely infected leaves using a modified Horsfall–Barratt scale,^31^ where 0%=1, 1–3%=2, 3–6%=3, 6–12%=4, 12–25%=5, 25–50%=6, 50–75%=7, 75–87%=8, 87–94%=9, 94–97%=10, 97–100%=11 and 100% dead tissue=12. Plants were scored in the field using the same scale as used in the greenhouse, except total foliage of whole plots was rated rather than the severity of infected leaves.

### Statistical analyses

ROS production in each genotype was calculated by adding all RLUs measured with the luminometer over the entire 15 cycles (for an hour). For each peptide, results for four leaf disks from each genotype were used from three independent experiments with three replications each were measured and least squares means of each were used for further analysis. For ROS analysis, RLUs measured from non-responsive leaf samples were deleted.

Area under the disease progress curve (AUDPC) was calculated based on weekly disease severity assessments. AUDPC is a quantitative summary of disease severity over time and compares average disease severity between pairs of adjacent time points.^32^ The AUDPC is calculated as follows:

$$AUDPC=\overset{n-1}{\underset{i=1}{\sum }}\frac{y_{i}+y_{i+1}}{2}\times (t_{i+1}-t_{i}),$$

where *y_i_* is the assessment of the disease at the *i*th observation, *t_i_* is the time at the *i*th observation and *n* is the total number of observations.

Analyses of variance were performed on ROS and AUDPC disease scores from the greenhouse and the field data using GLM procedure in Statistical Analysis Software (SAS v. 9.4, SAS Institute, Cary, NC, USA).^33^ Least squares of means of ROS and AUDPC were calculated using greenhouse and field data and least squares of means were separated from each genotypes using least significant difference value at 5% probability level. Correlation analysis was performed using the Pearson method in SAS 9.4.^33^

## Results

### Response of tomato to BS

Sixty-three tomato genotypes with diverse genetic backgrounds ranging from advanced breeding lines to wild relatives of tomato (Table S1) showed a broad range of response to inoculation with *Xanthomonas perforans*. AUDPC scores, normalized to a weekly basis, ranged from 18.5 for PI114490 to 34.9 for NC84173 based on BS symptoms (Table 1). There was a significant difference (*P*<0.05) between these tomato lines for response to BS when averaged over the two experiments (Table 1). *S. lycopersicum* var. *cerasiformae*, PI114490-1-1 was least infected by BS, whereas NC84173 showed the most severe BS symptoms. Lines showing the highest resistance (least disease severity) were Favorite, G357-2, NC50-7 and Fla8233. In contrast, lines Oxheart, 39BC-1, 48BC-1 and 71BC-1 had the greatest disease severity with the remainder being moderately infected (Table 1). Due to high disease pressure, fruits of most of the lines showed BS symptoms in the field trials in the summer of 2014 (data not shown).

### Responses of tomato lines to three PAMPs flg22, flg28-II and flg22-Xac

**Figure 1:**
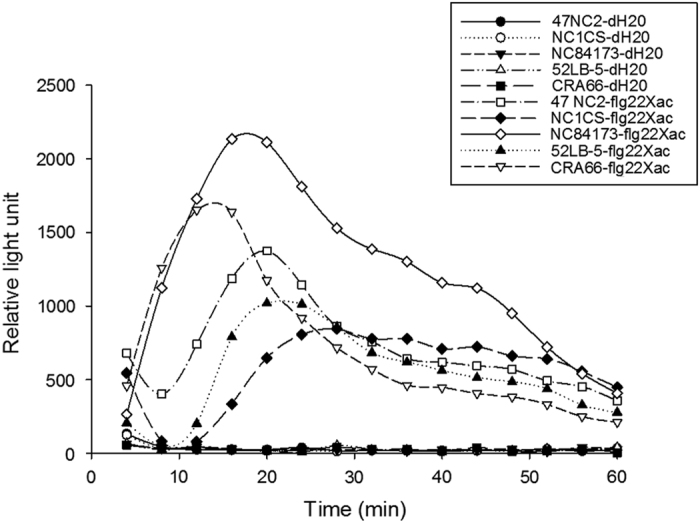
Production of reactive oxygen species (ROS) with and without flg22*-Xac*. When flg22*-Xac* was not used, distilled water (dH_2_O) was used to equalize the total volume in the 96-well plate. ROS production (in relative light units, RLUs) measured over 15 cycles (each cycle ~4 min, for a total of 60 min) in select tomato lines in response to the *Xanthomonas*-specific flagellin 22 (flg22*-Xac*; indicated by flg22*-Xac* at the end of tomato line name) or distilled water (indicated by dH_2_O at the end of tomato line name). Values and the separation of means based on least square means (LS means) values from analysis of variance (ANOVA) are detailed in Table 1. Data are the average of three biological replicates and four leaf disks from greenhouse plants.

To verify correlation of ROS production with PAMP activity, ROS was measured for 15 cycles (60 min) in five tomato lines with and without flg22*-Xac*. The effect of flg22*-Xac* was very distinct in those lines (Figure 1). Genotypes CRA66 and NC84173 showed peaks 12 and 16 min after treatment, respectively, with a steady decline thereafter until 60 min. Other genotypes 52LB-5, NC 1CS and 47NC2 showed a decrease until 10 min, followed by a peak at ~20, 20 and 28 min, respectively. All these lines showed a steady decline thereafter until 60 min. Although similar ROS production trends were found in all genotypes, genotypic differences in the timing of responses to PAMP treatment are clearly present. There was a significant difference (*P*<0.05) between all 63 tomato genotypes in ROS production (Table 1), as well as wide variation in levels of ROS production by each genotype when different PAMP peptides were used to initiate the oxidative burst (Table 1). For example, ROS production ranged from 1982 RLUs for 48BC-4R(96) to 49085 RLUs for NC50-7, from 2813 RLUs for 97E-1W(95) to 39302 RLUs for IRAT-L3 and from 844 RLUs for Yellow Pear to 144 336 RLUs for 72E-1(96) when peptides flg22*-Xac*, flg22 and flgII-28, respectively, were used (Table 1).

Each breeding line responded differently to the different peptides. For instance, the breeding line IRAT-L3, showed the highest ROS production in response to flg22, high ROS production in response to flg22*-Xac*, but a low response to flgII-28. In contrast, breeding lines 72E-1(96) and NC50-7 produced the highest amount of ROS in response to flgII-28 and flg22*-Xac*, respectively. None of these lines generated significant ROS in response to flg22. This trend (or lack thereof) is shown by the minimal correlation between ROS production in response to the different flagellin peptides (Table 1).

Likewise, responses to specific peptides also varied widely by genotype. For instance, ROS production in response to flg22*-Xac* (Table 1) was very high for NC50-7, NC109, HI7998 and 74L-1W (2008), whereas response to the same peptide was low for lines 97E-3W, Stupice, 48BC-1 and 39BC-1. Interestingly, BS disease scores of the flg22*-Xac* responsive lines NC50-7, NC109, HI7998 and 74L-1W (2008) were low, whereas the disease scores for the least responsive lines 97E-3W, Stupice, 48BC-1 and 39BC-1 were high (Table 1). Although not huge, a significant negative correlation between BS disease and total and maximum ROS production in response to flg22*-Xac* (−0.25 and −0.27, respectively) was observed (*P*=0.05; Table 1). Finally, although there was a wide variation in production of ROS in response to flg22 and flgII-28, there was no correlation with BS disease scores (Table 2).

### Correlation analysis

**Figure 2:**
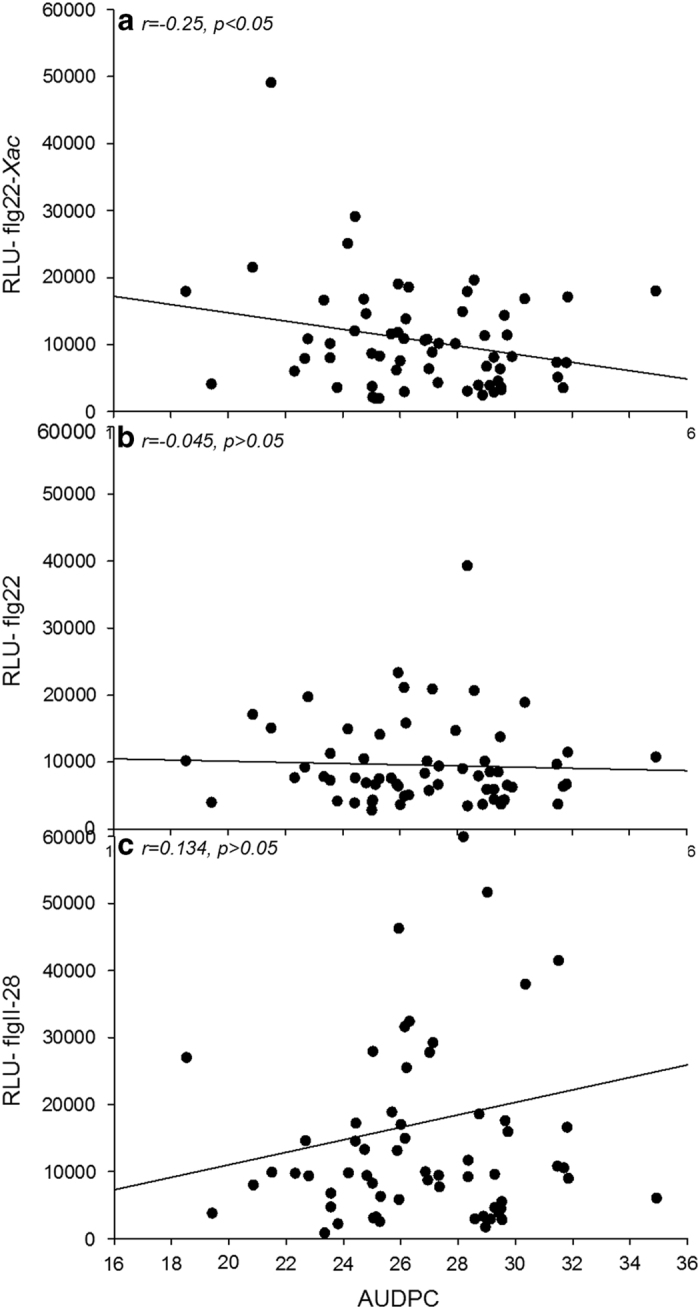
Correlation between area under disease progress curve (AUDPC) scores and relative light units (RLUs) used to assess reactive oxygen species (ROS) production in response to (**a**) *Xanthomonas*-specific flagellin 22 (flg22*-Xac*) (**b**) flagellin 22 (flg22) and (**c**) flagellin II-28 (flgII-28). AUDPC scores were calculated using Horsfall–Barratt scale and ROS (in RLUs) was measured over 15 cycles (each cycle ~4 min) for all tomato lines in this study. ROS was measured in a luminometer as described in Materials and Methods.

There were non-significant correlations between AUDPC and flg22 (*r*=0.04, *P*>0.05), and flgII-28 (*r*=0.13, *P>*0.05), respectively. However, there was a significant negative correlation between AUDPC and flg22*-Xac* (*r*=−0.25, *P*<0.05) (Table 2). A positive correlation was found between flg22 and flg22*-Xac* (*r*=0.41, *P*<0.05); however, there was no significant correlation between flgII-28 and the other two peptides (flg22 and flg22*-Xac*; Table 2). Correlations between BS disease development in tomato lines and RLUs as observed with the flg22*-Xac* assays are shown in Figure 2a. Non-significant correlations are shown in Figures 2b and c.

## Discussion

By screening such a large number of diverse tomato lines, this study identified plants with a broad range of responses to BS caused by *X. perforans* race T4. As expected, these results served to validate previous observations. For instance, line PI114490-1-1 that showed the lowest symptom development in this experiment had previously been reported to be BS resistant to race 2,^34^ whereas NC84173, a known susceptible line, showed extensive disease development. Line 74L-1W (2008) that has known resistance for BS (unpublished data) from *S. pimpinellifolium* L3707, a wild relative of tomato, was also resistant here, as were lines NC50-7 and NC109 (unpublished data). Likewise, HI7998 had high resistance in this study and was previously reported to have an hypersensitive response to *X. euvesicatoria*.^34^

Although we observed a wide range of responses to the three PAMPs by the 63 tomato lines used here, perhaps not surprisingly, there was no correlation between flg22 and flgII-28, resultant ROS production and BS disease severity (caused by *X. perforans*). Interestingly, a previous study showed that natural variation in heirloom tomato lines assessed had no correlation between the PAMPs used in that study and bacterial speck caused by *P. syringae* pv. *tomato*.^25^ In contrast, there was a small but statistically significant negative correlation (*r=−*0.25, *P*=0.05) between flg22*-Xac*-induced ROS production and BS disease scores in the present study, suggesting that there was the recognition of the role of flg22-*Xac* in defense response in tomato plants when they are attacked by the causal agent of BS (by *X. campestris*). Because the correlation was relatively small, this suggests there is a differential responsiveness to flg22-*Xac* among the various tomato lines in this relatively large group of tomato lines (that is, some lines show a larger response to the flg22-*Xac*, and at the same time are very resistant to the BS and vice versa). From this pool, we should be able to develop a population using high responsive and less responsive lines for the identification and mapping of PTI loci associated with BS resistance. Further, the fact that flg22*-Xac* is synthesized from a flagellin sequence from a bacteria of the same genus that causes BS suggests that it might produce a more relevant defense response in terms of ROS production and thus be more useful for screening tomato lines for defense response and identifying PTI loci. The observation that a PAMP from the same genus as the disease causing organism in question is able to elicit ROS production in tomato could form the basis of a technique for developing horizontal resistance to *Xanthomonas.* This is the first report to the authors’ knowledge of significant correlation between *Xanthomonas-*specific flagellin-induced ROS production and BS resistance in tomato. A significant correlation among a large set of lines derived from a wide genetic background as used here suggests that response to flg22*-Xac* may be encoded by a single loci, and as such likely warrants further efforts to map the loci. Lending further credence to this idea is the report that transferring EFR, a PAMP receptor from the cruciferous plant *A. thaliana* confers responsiveness to EF-Tu,^7^ led to an increase in resistance to a wide range of phytopathogenic bacteria in solanaceous crops such as *N. benthamiana* and *S. lycopersicum*.

Although their sequences were obtained from different sources,^19,20,29^ a significant positive correlation (*r*=0.41, *P*<0.05) between flg22 and flg22*-Xac* indicated that they are likely to be allelic to each other, being separated by only a few amino acids. The flg22 has already been reported to be highly active in many plants,^19^ which may have common sequences with the above peptides. Although flg22-*Xac* showed a significant correlation with BS, further investigation is needed to understand the association at molecular level.

The perception of flg22 has been reported to be mediated by the PRR FLS2.^19,35^ In yet another study, a separate PRR, FLS3 has been hypothesized to perceive flgII-28 in solanaceous plants.^20^ Further, the brassinosteroid receptor like-kinase BAK1 has been reported to interact with FLS2 to trigger ROS production in *A. thaliana* and *N. benthamiana*.^36,37^ Being an allelic variant of the *P. aeruginosa* flg22, derived from *Xanthomonas axonopodis* pv. *citri* flagella, flg22*-Xac*, it was found to induce defense response to BS in tomato suggests that the recognition of the flagellin sequence in tomato may be similar to that of PRR FLS2. Here the significant correlation between the ROS response to flg22*-Xac* and BS resistance suggests a means to study the specific PRR in tomato that mediates the defense response to *Xanthomonas* flagellin. Further studies on the PRR from lines showing high ROS response to the BS pathogen and introgression into tomato lines with good horticultural traits would expedite not only cultivar development but also mapping the flg22*-Xac* locus.

## Figures and Tables

**Table 1:** Mean comparison of tomato genotypes using AUDPC scores, and RLU production in response to flg22, flgII-28 and flg22*-Xac* peptides

*Genotype*	*AUDPC*	*flg22*	*flgII-28*	*flg22-Xac*
081-12-1X-gsms	25^b–g^	4338^b^	3075^b^	2163^b^
15BC-4(94)	26^a–g^	5076^b^	32 403^b^	18 582^b^
16BC-1(94)	29^a–e^	5932^b^	51 663^b^	6771^b^
16BC-2(94)	29^a–e^	6554^b^	15 949^b^	11 430^b^
17BC-1(94)	30^a–e^	6250^b^	78 916^a,b^	8220^b^
30LB-1W(95)	23^b–g^	9254^b^	14 599^b^	7931^b^
31LB-1W(95)	27^a–g^	5744^b^	27 784^b^	6409^b^
38BC-1(96)	30^a–e^	3717^b^	5513^b^	3651^b^
38BC-2R(96)	28^a–f^	34 612^b^	11 682^b^	3052^b^
39BC-1(96)	32^a,b^	6371^b^	10 553^b^	3559^b^
45LB-1(98)	30^a–e^	137 529^b^	4444^b^	6378^b^
46BC-2R(96)	26^a–g^	3655^b^	17 035^b^	7587^b^
47NC2	27^a–g^	8337^b^	9972^b^	10 635^b^
48BC-1(96)	32^a–c^	3737^b^	41 483^b^	5148^b^
48BC-1R(96)	26^a–g^	4928^b^	14 966^b^	2977^b^
48BC-3R(96)	25^b–g^	6653^b^	3234^b^	1999^b^
48BC-4R(96)	25^b–g^	7515^b^	2546^b^	1982^b^
52LB-1(98)	24^b–g^	11 291^b^	6757^b^	10 152^b^
52LB-2(98)	25^b–g^	4093^b^	27 911^b^	3764^b^
52LB-3(98)	27^a–g^	10 145^b^	8734^b^	10 806^b^
52LB-4(98)	29^a–e^	7921^b^	18 566^b^	3965^b^
71BC-1(96)	32^a–c^	9660^b^	10 798^b^	7356^b^
72E-1(96)	28^a–f^	14 696^b^	144 336^a^	10 153^b^
74L-1W(2008)	25^b–g^	10 518^b^	13 290^b^	16 793^b^
87E-1W(95)	26^a–g^	23 344^a,b^	46 285^b^	11 840^b^
89E-1W(95)	26^a–g^	6426^b^	5825^b^	19 058^b^
97E-1W(95)	25^b–g^	2813^b^	8254^b^	8689^b^
97E-2W(95)	24^b–g^	7279^b^	4719^b^	8032^b^
97E-3W(95)	29^a–e^	8547^b^	2914^b^	3917^b^
Aker's West Virginia	30^a–d^	18 895^a,b^	37 970^b^	16 835^b^
Black from Tula	26^a–g^	15 810^b^	25 497^b^	13 835^b^
Brandywine	27^a–g^	20 907^a,b^	29 236^b^	8895^b^
Cherokee Purple	29^a–e^	5934^b^	9591^b^	8120^b^
CRA66	29^a–e^	10 141^b^	1719^b^	11 337^b^
Favorite	19^g^	4008^b^	3799^b^	4118^b^
FD502-3-Bk	29^a–e^	3690^b^	3318^b^	2481^b^
Fla7600	24^b–g^	4169^b^	2201^b^	3616^b^
Fla8000	30^a–e^	4160^b^	2824^b^	3278^b^
Fla8233	22^c–g^	7676^b^	9740^b^	6044^b^
G357-1(2011)	23^b–g^	19 721^a,b^	9343^b^	10 863^b^
G357-2(2011)	21^e–g^	17 092^a,b^	7992^b^	21 538^b^
HI7981	24^b–g^	3902^b^	14 532^b^	12 048^b^
HI7997	29^a–f^	20 670^a,b^	2940^b^	19 639^b^
HI7998	24^b–g^	7640.^b^	17 216^b^	29 113^a,b^
IRAT-L3	28^a–f^	39 303^a^	9219^b^	17 924^b^
Moneymaker	26^a–g^	21 109^a,b^	31 609^b^	10 886^b^
NC109	24^b–g^	14 932^b^	9796^b^	25 086^a,b^
NC123S	29^a–e^	8525^b^	3858^b^	45 656^b^
NC161L	25^b–g^	14 111^b^	6284^b^	8299^b^
NC22L-1(2008)	32^a,b^	6720^b^	16 610^b^	7305^b^
NC2CELBR	26^a–g^	6748^b^	13 148^b^	6195^b^
NC50-7	22^d–g^	15 083^b^	9903^b^	49 085^a^
NC84173	35^a^	10 762^b^	6050^b^	18014^b^
NCEBR-6	27^a–g^	9405^b^	7699^b^	10181^b^
NCEBR-8	30^a–e^	4359^b^	17 583^b^	14 367^b^
Orange Strawberry	27^a–g^	6663^b^	9420^b^	4325^b^
Oxheart	32^a,b^	11 484^b^	8996^b^	17 119^b^
PI114490-1-1	19^g^	10 187^b^	27 016^b^	17 938^b^
PI134417	28^a–f^	9027^b^	59 944^b^	14 932^b^
Rutgers	25^b–g^	6907^b^	9412^b^	14 622^b^
Stupice	30^a–e^	4424^b^	4627^b^	2915^b^
Yellow Pear	23^b–g^	7832^b^	844^b^	16 644^b^
Yellow Stuffer	26^b–g^	7605^b^	18 883^b^	11 597^b^
LSD(0.05)	1.1	2275.2	2811.8	7910.2

Data are the average of three biological replicates and four leaf disks from greenhouse plants.

LSD, least significant difference at 5% probability level. Superscripts are used to indicate genotypes having significantly different AUDPC (BS resistance) and PAMP response. Figures followed by the same letter in each column do not differ significantly from each other, whereas figures followed by a different letter(s) differ significantly from each other. Genotypes can also be grouped based on letters for AUDPC (BS resistance) and PAMP response.

**Table 2:** Correlation between bacterial spot disease (AUDPC) severity and ROS production in tomato

*Variable*	*AUDPC*	*flg22*	*flgII-28*
flg22	−0.04^NS^		
flgII-28	0.13^NS^	0.13^NS^	
flg22*-Xac*	−0.25*	0.41**	0.04^NS^

Abbreviation: AUDPC, area under disease progress curve.

Correlation analysis between reactive oxygen species production from a luminol-based oxidative burst assay using the PAMPs flagellin 22 (flg22), flagellin II-28 (flg28) and *Xanthomonas*-specific flagellin 22 (flg22*-Xac*) to generate the ROS and bacterial spot disease severity (measured as AUDPC) produced by inoculation with a *Xanthomonas perforans*, race T4, field isolate.

^NS^, * and ** are non-significant, significant at *P* value <0.05, and 0.01, respectively.
