# Supplementary material for: Differential response of tomato genotypes to Xanthomonas-specific pathogen-associated molecular patterns and correlation with bacterial spot (Xanthomonas perforans) resistance
Source: Hortic Res. 2016 Aug 10;3:16035–. doi: 10.1038/hortres.2016.35 (PMC4978809; doi:10.1038/hortres.2016.35)
Supplement: Supplementary Table S1 [file hortres201635-s1.doc]

**Supplemental Table S1**

List of tomato lines, description and source, used in Bacterial spot disease screening and reactive oxygen species production assays.

| **Genotype** | **Scientific name** | **Comment** | **Source** |
| --- | --- | --- | --- |
| NC714 | *Solanum lycopersicum* | Breeding line | NCSU |
| 081-12-1X-gsms | *S. lycopersicum* | Breeding line | NCSU |
| 15BC-4(94) | *S. lycopersicum* | Breeding line | NCSU |
| 16BC-1(94) | *S. lycopersicum* | Breeding line | NCSU |
| 16BC-2(94) | *S. lycopersicum* | Breeding line | NCSU |
| 17BC-1(94) | *S. lycopersicum* | Breeding line | NCSU |
| 30LB-1W(95) | *S. lycopersicum* | Breeding line | NCSU |
| 31LB-1W(95) | *S. lycopersicum* | Breeding line | NCSU |
| 38BC-1(96) | *S. lycopersicum* | Breeding line | NCSU |
| 38BC-2R(96) | *S. lycopersicum* | Breeding line | NCSU |
| 39BC-1(96) | *S. lycopersicum* | Breeding line | NCSU |
| 45LB-1 | *S. lycopersicum* | Breeding line | NCSU |
| 46BC-2R(96) | *S. lycopersicum* | Breeding line | NCSU |
| 47NC2 | *S. lycopersicum* | Breeding line | NCSU |
| 48BC-1(96) | *S. lycopersicum* | Breeding line | NCSU |
| 48BC-1R(96) | *S. lycopersicum* | Breeding line | NCSU |
| 48BC-3R(96) | *S. lycopersicum* | Breeding line | NCSU |
| 48BC-4R(96) | *S. lycopersicum* | Breeding line | NCSU |
| 52LB-1 | *S. lycopersicum* | Breeding line | NCSU |
| 52LB-2 | *S. lycopersicum* | Breeding line | NCSU |
| 52LB-3 | *S. lycopersicum* | Breeding line | NCSU |
| 52LB-4 | *S. lycopersicum* | Breeding line | NCSU |
| 52LB-5 | *S. lycopersicum* | Breeding line | NCSU |
| 71BC-1(96) | *S. lycopersicum* | Breeding line | NCSU |
| 72E-1(96) | *S. lycopersicum* | Breeding line | NCSU |
| 74L-1W(2008) | *S. lycopersicum* | Breeding line | NCSU |
| 87E-1W(95) | *S. lycopersicum* | Breeding line | NCSU |
| 89E-1W(95) | *S. lycopersicum* | Breeding line | NCSU |
| 97E-1W(95) | *S. lycopersicum* | Breeding line | NCSU |
| 97E-2W(95) | *S. lycopersicum* | Breeding line | NCSU |
| 97E-3W(95) | *S. lycopersicum* | Breeding line | NCSU |
| Akers West Virginia | *S. lycopersicum* | Heirloom |  |
| Black from Tula | *S. lycopersicum* | Heirloom |  |
| Brandywine | *S. lycopersicum* | Heirloom |  |
| CRA66 | *S. lycopersicum* | Breeding line | France |
| Cherokee Purple | *S. lycopersicum* | Heirloom |  |
| FD502-3-BK | *S. lycopersicum* | Breeding line | NCSU |
| Favorite | *S. lycopersicum* | Breeding line |  |
| Fla7600 | *S. lycopersicum* | Breeding line | UFL |
| Fla8000 | *S. lycopersicum* | Breeding line | UFL |
| Fla8233 | *S. lycopersicum* | Breeding line | UFL |
| G357-1(2011) | *S. lycopersicum* | Breeding line |  |
| G357-2(2011) | *S. lycopersicum* | Breeding line |  |
| HI7981 | *S. lycopersicum* | Breeding line | UFL |
| HI7997 | *S. lycopersicum* | Breeding line | UFL |
| HI7998 | *S. lycopersicum* | Breeding line | UFL |
| IRAT-L3 | *S. lycopersicum* | Breeding line | France |
| Moneymaker | *S. lycopersicum* | Heirloom |  |
| NC109 | *S. lycopersicum* | Breeding line | NCSU |
| NC123S | *S. lycopersicum* | Breeding line | NCSU |
| NC161L-1W(2007) | *S. lycopersicum* | Breeding line | NCSU |
| NC22L-1(2008) | *S. lycopersicum* | Breeding line | NCSU |
| NC2CELBR | *S. lycopersicum* | Breeding line | NCSU |
| NC 1CS | *S. lycopersicum* | Breeding line | NCSU |
| NC50-7 | *S. lycopersicum* | Breeding line | NCSU |
| NC84173 | *S. lycopersicum* | Breeding line | NCSU |
| NCEBR-6 | *S. lycopersicum* | Breeding line | NCSU |
| NCEBR-8 | *S. lycopersicum* | Breeding line | NCSU |
| Orange Strawberry | *S. lycopersicum* | Heirloom |  |
| Oxheart | *S. lycopersicum* | Heirloom |  |
| PI114490-1-1 | *S. lycopersicum var. cerasiforme* | Wild | USDA |
| PI134417 | *S. habrachaites* | Wild | USDA |
| Rutgers | *S. lycopersicum* | Breeding line | Rutgers |
| Stupice | *S. lycopersicum* | Heirloom |  |
| Yellow Pear | *S. lycopersicum* | Heirloom |  |
| Yellow Stuffer | *S. lycopersicum* | Heirloom |  |
